# Effects of Visual Input on Postural Stability and Compensatory Strategies in Adults with Chronic Low Back Pain

**DOI:** 10.3390/vision9010014

**Published:** 2025-02-20

**Authors:** Paul S. Sung, Dongchul Lee

**Affiliations:** 1 Doctor of Physical Therapy Program in the School of Health Sciences, Indiana Wesleyan University, Marion, IN 46953, USA; 2 Neurostim Insight, Santa Clarita, CA 91390, USA; dreamittogether@gmail.com

**Keywords:** low back pain, center of pressure, normalized stability index, visual condition, posture, postural sway, contralateral toe touch, balance, posture compensation, proprioception

## Abstract

Chronic low back pain (LBP) impairs balance control due to deficits in sensory integration, yet limited research examines postural sway under varying visual conditions. This study assessed the effects of visual input on postural stability using the normalized stability index, sway excursions, and contralateral toe-touch durations during repeated one-leg standing tasks. Thirty-two adults with LBP and 40 control subjects performed dominant limb standing on a force plate. Outcome measures included the Oswestry disability index, visual analog scale, normalized stability index, sway excursions (anteroposterior [AP], mediolateral [ML]), and contralateral toe-touch duration. The LBP group showed a significant interaction for the normalized stability index under visual conditions (F = 4.95, *p* = 0.03) with reduced stability in the second trial of the eyes-open condition (t = 1.71, *p* = 0.04). Sway excursions increased in the AP direction during the first trial (t = −2.43, *p* = 0.01) and in the ML (t = −2.09, *p* = 0.02) and AP (t = −1.84, *p* = 0.03) directions during the third trial. Contralateral toe-touch duration increased in the second trial (t = −2.06, *p* = 0.02). Individuals with LBP exhibited balance deficits, particularly under eyes-open conditions, relying on compensatory strategies. Optimizing neuromuscular control and sensory integration may improve postural stability.

## 1. Introduction

Postural stability represents the ability of the postural control system to maintain the center of pressure (COP) within the feet contact area during upright standing [1]. This system integrates various sensory information (visual, proprioceptive, vestibular, and somatosensory) and provides motor responses acting as biomechanical strategies and neuromuscular adaptations to maintain balance [2]. Recent studies on healthy individuals using baropodometry have evaluated postural adaptations under different visual conditions, such as eyes-open and eyes-closed [2,3].

Chronic low back pain (LBP) is a leading cause of disability worldwide, affecting older adults and significantly limiting functional mobility [4]. One of the key consequences of LBP is impaired balance control, which increases fall risk and reduces overall stability [5]. Individuals with LBP often exhibit deficits in sensory integration, particularly in proprioception and vestibular function, leading to altered postural control strategies and increased postural sway [6,7]. These postural deficits disrupt stability, further elevating fall risk and impairing mobility [8,9].

While studies have demonstrated that the integration of visual, proprioceptive, and vestibular inputs plays a crucial role in balance control in healthy individuals, the compensatory strategies used by individuals with LBP under altered visual conditions remain inconsistent in the literature [8,9]. This variability underscores the need for further research to refine balance assessment methods and explore postural strategies during one-leg standing tasks. Impaired proprioception is a major contributing factor to these balance deficits, often causing individuals with LBP to over-rely on visual input, which can exacerbate instability when visual cues are unavailable [10,11,12]. In fact, proprioceptive feedback from ankle receptors is essential for postural and balance control in both static and dynamic conditions [13].

Balance disorders become increasingly prevalent with age and are often associated with LBP, adding complexity to the clinical presentation [14]. Increased postural sway in individuals with LBP frequently reflects a reliance on adaptive strategies, such as toe touches, to maintain stability. While light touch and contralateral limb support have been shown to enhance somatosensory feedback and reduce sway, the most effective compensatory strategies for individuals with chronic LBP remain unclear [15,16]. Understanding how visual input and compensatory mechanisms influence sway and balance may inform the development of targeted rehabilitation strategies.

Previous studies suggest that individuals with LBP face challenges maintaining balance under altered visual conditions due to impaired sensory integration and compensatory strategies [17,18,19]. They often demonstrate increased variability in lumbar movements and decreased stability in response to perceived postural threats, highlighting how pain and fear of movement influence motor behavior [20,21,22]. While meta-analyses have reported inconsistent findings on the role of visual input in postural stability, high heterogeneity and methodological biases in balance assessments may have contributed to these discrepancies [23]. A more comprehensive approach that integrates visual input, sway control, and neuromuscular responses is essential to minimize bias and improve the accuracy of balance evaluations.

In our study, we utilized a single leg standing task, which is a widely recognized test for assessing postural control and functional mobility [24,25,26]. We examined the normalized stability index, sway excursions, and contralateral toe-touch durations during dominant limb standing in individuals with and without LBP. The stability index, incorporating time-in-boundary (TIB) measures, evaluates sway in both the anteroposterior (AP) and mediolateral (ML) directions, offering a detailed assessment of postural control within center of pressure (COP) thresholds [27,28]. These metrics are particularly useful in repeated trials of unilateral standing, where COP sway ranges serve as key indicators of balance stability. However, research suggests that COP spatial-based measures have limited sensitivity, often overlooking temporal aspects of balance control during dynamic activities [29,30].

Maintaining postural stability is essential for daily activities that require single-limb support. Individual characteristics, such as age, body mass index (BMI), limb dominance, and gender, can influence compensatory postural stability, potentially affecting the generalizability of study outcomes [31,32]. Therefore, our study aims to compare normalized stability indices, AP and ML sway excursions, and contralateral toe-touch durations while considering visual input between older adults with and without LBP during repeated one-leg standing tasks. We hypothesize that individuals with LBP will exhibit decreased stability and increased sway excursions, particularly in eyes-closed conditions, compared to control subjects.

## 2. Methods

### 2.1. Participants

Subjects were recruited from the community through targeted advertising. Those who met the inclusion criteria were informed in detail about the research aims and procedures. All subjects provided written informed consent, and the study adhered to Institutional Review Board guidelines (IRB #1653.21). Eligibility for participation was determined based on the following criteria: (1) age between 50 and 85 years, (2) right limb dominant, (3) persistent LBP for a minimum duration of three months prior to data collection, (4) absence of severe pathologies, such as nerve root compromise, at the time of data collection, and (5) no existing visual impairments or conditions that would preclude unilateral standing.

Exclusion criteria included: (1) a diagnosed psychological disorder that could potentially disrupt the study protocol, (2) manifest neurological symptoms, including sensory deficits or motor paralysis, and/or (3) pregnancy. Older adults in the control group were considered based on age, gender, and BMI to minimize anthropometric variations. The study focused on the dominant sides of the upper and lower limbs, given their potential influence on balance outcomes [33,34]. To ensure a consistent and standardized approach in minimizing limb dominance effects on sway measurements, the right lower limb was designated as the dominant side for all subjects based on their self-reported preference for using the right limb to kick a ball [35,36].

### 2.2. Experimental Procedures

Upon arrival at the laboratory, subjects completed health status questionnaires that included demographic information. The level of disability was measured using the Oswestry disability index (ODI), which is a 10-item questionnaire that evaluates the impact of low back (or leg) pain on daily activities [37]. In addition, pain intensity was assessed using a visual analog scale (VAS), which allows subjects to mark their pain level on a continuous line, providing both continuous and interval-level measurement data [38]. The magnitude of improvement observed exceeded the minimal clinically important difference, indicating a clinically meaningful enhancement in functional disability. These tools offer valuable insights into a patient’s perceived disability and pain levels [39]. Therefore, incorporating specific balance assessments is essential to comprehensively understand the effects of LBP on an individual’s functional capabilities.

Subjects stood barefoot on a calibrated force plate with the medial malleolus aligned to a horizontal guideline and the calcaneus aligned with the transverse axis. The y-axis measured AP movements, while the x-axis recorded ML movements. Postural stability was assessed using the Bertec Balance Advantage^®^ system for Computerized Dynamic Posturography with Immersion Virtual Reality (CDP-IVR) (BERTEC, Columbus, OH, USA), which monitored balance performance and postural adjustments [28,40,41,42].

Subjects performed dominant limb standing tasks under two visual input conditions: eyes open and eyes closed. Each task involved standing on one leg for 10 s with the contralateral knee flexed to approximately 30 degrees. Arms were initially positioned at the sides, but compensatory arm movements were permitted to maintain dynamic balance. All tasks were demonstrated by the investigator to ensure consistency. A safety harness was used to prevent falls. Kinetic data were collected using the calibrated force platform Bertec Type 4060 (Columbus, OH, USA) at a sampling frequency of 1000 Hz [43], and the data were filtered using a fourth-order low-pass Butterworth filter with a 12 Hz cutoff frequency [44,45]. All measurements were normalized to individual body weight, and zero offsets were determined using unloaded platform measurements. The manufacturer-calibrated force plate included a sensitivity matrix to convert voltages into forces and torques accurately. Changes in the COP during unilateral stance balance tasks were recorded, reflecting postural stability. The force plates demonstrated moderate to very high reliability across various postural sway measures [46], ensuring robust data for analysis.

The normalized stability index was based on the TIB metric, which quantifies the percentage of time the COP remains within a predefined boundary. TIB emphasizes proactive stability, offering insight into the ability to maintain controlled balance. The predefined threshold ellipse (radius = 25 mm) standardizes comparisons of postural control. The metrics we utilized in our data analyses are detailed in our previous study [22].

**Figure 1:**
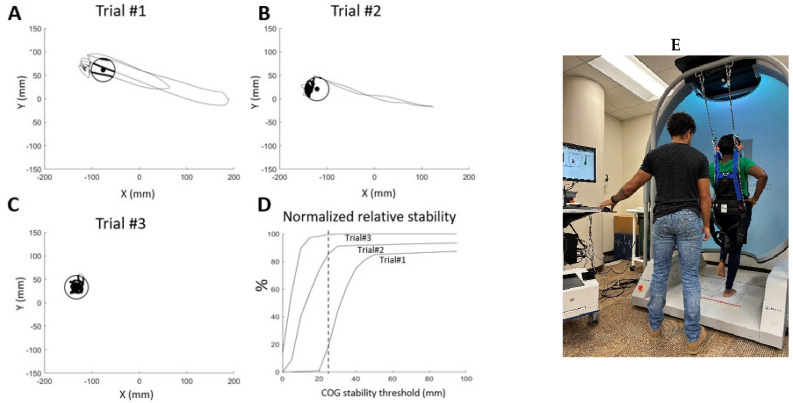
An example of the calculation of the normalized stability index (%) using center of pressure (COP) data during dominant limb standing under eyes-open conditions. Data points within the threshold ellipse were plotted to illustrate stability differences between trials. Trial #1 (**A**), the trajectory of the first trial is plotted for a subject with low back pain (LBP). The x-axis represents mediolateral (ML) positions, and the y-axis represents anteroposterior (AP) positions. Deviations from the trajectory’s central point (black dot) indicate challenges in maintaining unilateral standing. Data points within the threshold ellipse are represented by dark lines, highlighting time spent within the predefined stability boundary. Trials #2 and #3 (**B**,**C**), the second and third trials for the same subject with LBP are shown. In Trial #3, the COP trajectory predominantly remains within the threshold ellipse, suggesting improved relative stability compared to Trial #1. (**D**) The normalized relative stability time (%) is presented, representing the proportion of time the COP trajectory stays within a threshold ellipse during a 10 s standing period. Larger thresholds result in increased relative stability time. The vertical dotted line indicates an example threshold (25 mm), as used in (**A**–**C**). Subject A (a participant with LBP) achieved 18% stability tolerance, while Subject C (a participant without LBP) maintained 85% stability tolerance within the threshold, demonstrating significantly better postural control. (**E**) Initial setup for the unilateral standing test. A subject was secured in a full-body safety harness and instructed to stand barefoot on his/her dominant leg for 10 s, flexing the contralateral knee at an angle of approximately 30° while maintaining the standing limb vertically.

COP movements in AP and ML directions were analyzed, and Figure 1 illustrates a threshold ellipse (radius = 25 mm), representing the predefined spatial boundary for analyzing COP trajectories in subjects with and without LBP across three trials. The threshold was selected to standardize comparisons of postural control within a clinically meaningful range. The mathematical equations used to calculate the threshold ellipse and related insights into the stability of subjects by quantifying the consistency of the COP within pre-defined balance thresholds were obtained from a previous study [22]. The time series, commonly recorded in postural studies, were used to describe postural sway, and we calculated the median excursion in centimeters (cm). These parameters were obtained for the AP and the ML directions using equations described in the literature [47].

Contralateral toe-touch durations were quantified by analyzing the COP trajectory to identify instances when the non-stance foot (contralateral foot) contacted the ground or force plate during the standing task. The analysis involved calculating the COP distance from the center point on the force plate and applying a predefined boundary to detect toe-touch events. These events were characterized by COP displacements that exceeded the boundary and a sharp shift toward the contralateral side. The total duration of all detected toe-touch events during a trial was summed to derive the contralateral toe-touch duration. To ensure the accuracy of these measurements, the COP trajectory was visually inspected and, where possible, cross-referenced with video recordings to confirm the occurrence and timing of toe-touch events. This method provided a reliable assessment of compensatory toe-touch behavior during balance tasks. Our study utilized COP data analysis to evaluate postural control effectiveness under varying conditions, including changes in visual input across repeated trials. This analysis helps identify balance deficits and informs the use of contralateral toe-touches as compensatory mechanisms. The duration spent in stable positions indicates balance proficiency, with COP representing the point of applied ground reaction force. The distance (Dist) used to assess contralateral toe-touches was calculated using the following Formula (1):

(1) $$Dist=\sqrt{{ML}^{2}+{AP}^{2}}$$

COP movements were further analyzed to determine the total time spent in stable positions and to detect toe-touch events, reflecting compensatory mechanisms during balance tasks. Only data from subjects with three valid trials were included in the analysis. Kinetic variables were analyzed for AP and ML directions [47,48]. Mean and standard deviation values were computed for all sway metrics, providing a comprehensive assessment of postural stability under varying visual conditions.

### 2.3. Statistical Analysis

We conducted preliminary power analyses based on pilot data comparing groups, assuming a type I error rate of 0.05. In our study, the sample size calculation determined that a minimum of 29 subjects per group would provide 80% power to detect an effect size of 0.4 based on the method by Muller and Barton [49]. Effect sizes were confirmed by partial Eta-squared values (η^2^p) within repeated measures analysis of variance (ANOVA) squared (small ≥ 0.01, medium ≥ 0.06, large ≥ 0.14), which was used to indicate the mean difference between groups [50]. The independent variables included groups (with and without LBP).

To investigate differences in individual characteristics between groups, an independent *t*-test was utilized. In addition, mixed repeated measures ANOVA were conducted to analyze main and interaction effects on the normalized stability index, contralateral toe-touch durations, and sway excursions, while considering visual conditions. The general linear model was applied to assess all continuous dependent variables based on a by-group factorial experimental design.

In cases where demographic factors revealed group differences, they were included as covariates in the analysis. Failure to control such confounding variables could compromise the generalizability of the findings, particularly in adults with LBP. All statistical analyses were performed using SPSS version 28.0 (IBM Corp., Armonk, NY, USA) with a significance level set at 0.05 for all tests.

## 3. Results

As shown in Table 1, the study included 32 subjects with LBP (20 female and 12 male) and 40 control subjects (19 female and 21 male). There were no significant group differences in gender (χ^2^ = 1.61, *p* = 0.21), age (t = −1.46, *p* = 0.15), or BMI (t = 0.86, *p* = 0.39). However, the LBP group reported moderate disability levels with significantly higher ODI scores (t = −10.19, *p* = 0.001) and higher pain levels based on the VAS (t = −7.08, *p* = 0.001) compared to the control group.

**Figure 2:**
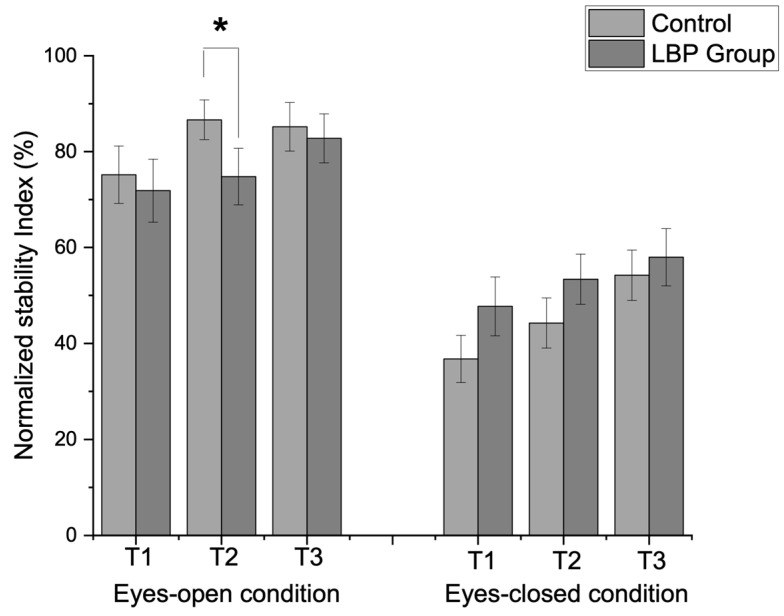
Results of the normalized stability index (%) differences between groups. The groups demonstrated a significant interaction on visual conditions (F = 4.95, *p* = 0.03), although there was no significant interaction on vision x repetitions (F = 1.11, *p* = 0.14). The LBP group demonstrated significantly decreased stability during the second trial (t = 1.71, *p* = 0.04) in the eyes-open condition. (LBP: low back pain, T: trial, * < 0.05).

The normalized stability index (%) was compared between groups, and the results are summarized in Figure 2. A significant group interaction was observed for visual conditions (F = 4.95, *p* = 0.03, η^2^p = 0.08), but no significant interaction was found for visual conditions × repetitions (F = 1.11, *p* = 0.14, η^2^p = 0.02). Significant main effects were observed for visual conditions (F = 95.91, *p* = 0.001, η^2^p = 0.62) and repetitions (F = 14.56, *p* = 0.001, η^2^p = 0.21). The LBP group demonstrated a significantly lower stability index during the second trial (t = 1.71, *p* = 0.04) in the eyes-open condition compared to the control group.

**Figure 3:**
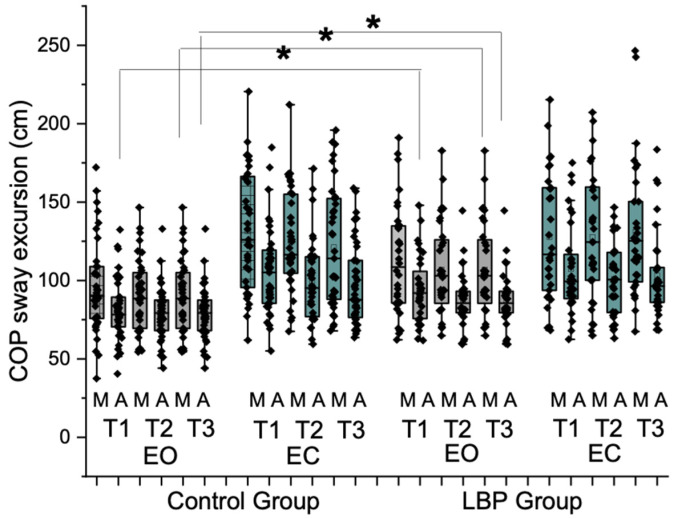
Results of sway excursions during repeated one-leg standing trials across groups under varying visual conditions. A significant interaction was observed between groups, visual conditions, and repetitions (F = 4.03, *p* = 0.04). The LBP group exhibited significantly increased sway excursion in the anteroposterior direction during the first trial (t = −2.43, *p* = 0.01) and in the mediolateral direction during the third trial (t = −2.09, *p* = 0.02) in the eyes-open condition. In addition, an increased sway in the anteroposterior direction was observed during the third trial (t = −1.93, *p* = 0.02). (LBP: low back pain, COP: center of pressure, M: mediolateral, A: anteroposterior, T: one-leg standing trial, EO: eyes-open condition, EC: eyes-closed condition (* < 0.05).

Sway excursions (cm) during repeated one-leg standing trials were compared between groups, accounting for visual conditions. The results, presented in Figure 3, revealed a significant interaction between groups, visual conditions, and repetitions (F = 4.03, *p* = 0.04, η^2^p = 0.06). However, there was no significant interaction for visual conditions × sway directions (F = 2.23, *p* = 0.14, η^2^p = 0.03). Significant main effects were observed for visual conditions (F = 50.06, *p* = 0.001, η^2^p = 0.42), repeated trials (F = 6.61, *p* = 0.01, η^2^p = 0.09), and sway directions (F = 51.75, *p* = 0.001, η^2^p = 0.43). Specifically, the LBP group demonstrated significantly increased sway excursion in the AP direction during the first trial (81.98 ± 19.30 for the control group vs. 94.26 ± 22.64 for the LBP group; t = −2.43, *p* = 0.01), increased sway in the ML direction during the third trial (95.01 ± 35.78 for the control group vs. 108.55 ± 31.20 for the LBP group; t = −2.09, *p* = 0.02), and increased sway in the AP direction during the third trial (79.84 ± 18.68 for the control group vs. 88.34 ± 17.16 for the LBP group; t = −1.93, *p* = 0.02) in the eyes-open condition.

**Figure 4:**
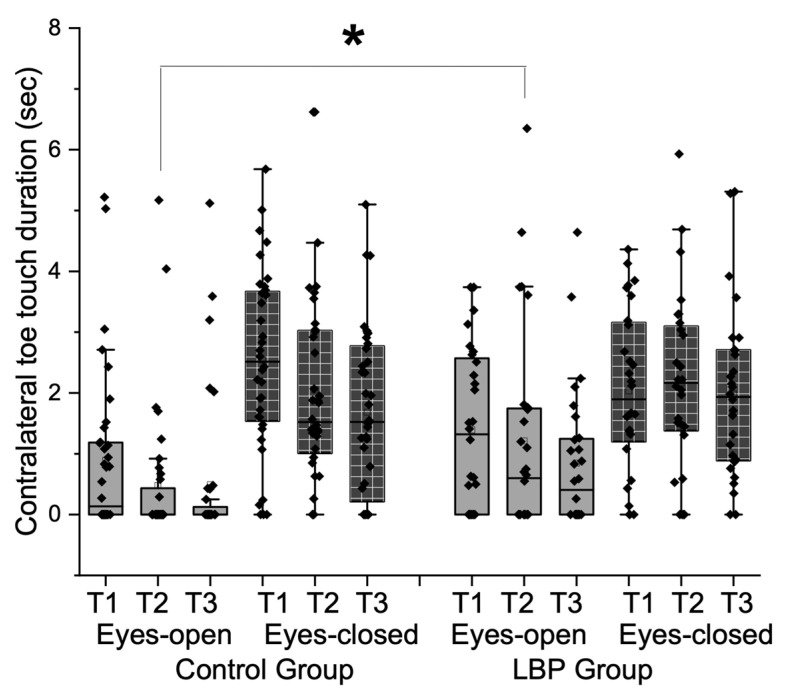
Box plots illustrate contralateral toe-touch duration across groups under different visual conditions and repeated trials. A significant interaction was observed between groups and visual conditions (F = 4.71, *p* = 0.03) as well as between visual conditions and repetitions (F = 7.23, *p* = 0.01). Specifically, during the second trial in the eyes-open condition, the LBP group exhibited significantly longer toe-touch durations compared to the control group (t = −2.06, *p* = 0.02) (LBP: low back pain, T: trial, * < 0.05).

The differences in sway excursions are closely related to contralateral toe-touch durations, as increased sway often necessitates compensatory strategies to maintain balance. Therefore, we compared the clinical relevance of these measures between groups while considering visual conditions (Figure 4) to highlight how sway patterns influence the need for toe-touch support. Significant group interactions were observed for visual conditions (F = 4.71, *p* = 0.03, η^2^p = 0.07) as well as visual conditions x repetitions (F = 7.23, *p* = 0.01 η^2^p = 0.11). There were main effect differences on visual conditions (F = 84.69, *p* = 0.001, η^2^p = 0.57) and repetitions (F = 20.51, *p* = 0.001, η^2^p = 0.25). A specific group difference was observed during the second trial in the eyes-open condition, where the LBP group exhibited significantly longer toe-touch durations compared to the control group (t = −2.06, *p* = 0.02).

## 4. Discussion

This study provides novel insights into postural stability in the LBP group, highlighting the unexpected finding that postural instability was more pronounced under eyes-open conditions rather than eyes-closed conditions. This contrasts with the traditional assumption that visual deprivation exacerbates postural deficits in LBP. The observed increased sway in both ML and AP directions during repeated trials under eyes-open conditions underscores the complex role of visual input in postural control. These results expand current understanding by suggesting that reliance on visual feedback may not compensate effectively for sensory integration deficits in the LBP group.

Our subjects with LBP reported moderate ODI and minimal pain level according to the VAS, as other studies have shown that chronic pain negatively impacts balance, which affects static, dynamic, and reactive balance capabilities [51,52,53]. The normalized stability index incorporates TIB measures to assess postural stability by quantifying the percentage of time the COP remains within a predefined spatial boundary. This metric provides a proactive perspective on balance control, emphasizing an individual’s ability to maintain stability within a controlled spatial range [22,40]. By evaluating the duration, the COP remains within these thresholds and TIB offers insights into postural stability and neuromuscular efficiency. In addition, sway excursions and contralateral toe-touch durations serve as critical metrics for assessing balance performance and compensatory strategies.

The normalized stability index in our study reflects the overall stability of an individual by quantifying the ability to maintain the COP within defined boundaries. Clinically, a higher stability index indicates greater postural instability, which may correlate with an increased risk of falls or impaired balance control. In contrast, previous time-to-boundary (TTB) measures have primarily been used to assess balance deficits by evaluating the time it takes for the COP to approach the edge of the base of support, reflecting the speed at which instability might occur without corrective action [54,55]. While TTB is valuable for assessing reactive stability and responses to destabilizing forces, it is less relevant for understanding static or prolonged stability within sway boundaries. Incorporating TIB into clinical assessments could offer a more comprehensive approach to evaluating postural control, enabling the development of targeted rehabilitation strategies. These strategies should aim to reduce reliance on compensatory mechanisms, such as contralateral toe-touch, and enhance neuromuscular coordination to improve overall balance and functional stability.

In our study, participants were selected with similar anthropometric characteristics to minimize potential confounding effects related to age and gender. There were no significant group differences in gender or BMI among our participants, which are factors known to influence postural stability [31,56,57]. However, the findings from these studies were based on small sample sizes, limiting their generalizability.

While previous studies have primarily focused on the effects of visual deprivation on balance [58,59], their findings addressed a critical gap by exploring how repeated exposure to balance tasks under varying visual conditions impacts postural stability. The identification of a ceiling effect under eyes-closed conditions, which limited group differentiation, suggests that traditional balance assessments may overlook subtle neuromuscular deficits observable under eyes-open conditions. In addition, the study highlights the role of contralateral toe-touch duration as a compensatory mechanism, which is a factor rarely examined in the context of LBP-related balance impairments.

In our study, the LBP group demonstrated increased reliance on visual feedback during balance tasks and frequently employed compensatory strategies, such as contralateral toe-touches, during dominant limb standing. The reduced stability in the LBP group during the second trial under the eyes-open condition may indicate a need for neuromuscular coordination training and interventions to enhance proprioceptive function to reduce reliance on visual feedback (Figure 2). When sway excursions were less pronounced, the observed increase in contralateral toe-touch durations revealed challenges in proprioceptive acuity and neuromuscular responses. This finding is consistent with previous research linking sway metrics to sensorimotor deficits [60]. Prior research highlights that sway excursions effectively indicate sensorimotor deficits [61,62] with visual input, emphasizing the context-specific nature of postural responses.

We hypothesized that individuals with LBP would exhibit decreased stability and increased sway excursions, particularly under eyes-closed conditions, compared to control subjects. However, this hypothesis was rejected. Contrary to our expectations, the LBP group demonstrated greater postural instability under eyes-open conditions. This finding aligns with studies highlighting the importance of visual target distance for accurately interpreting postural stability and plantar pressure parameters, which emphasizes the need to standardize target distance in balance assessments [2,63]. Interestingly, these results were not attributed to repetitive exposure to balance tasks, which typically enhances postural control plasticity by improving proprioceptive function and reducing reliance on visual input. Additionally, a potential ceiling effect observed under eyes-closed conditions may have limited our ability to detect group differences [64]. Our findings suggest that individuals with LBP experience more pronounced challenges in maintaining stable postures when subjected to repetitive neuromuscular demands.

Visual input significantly influenced postural stability, as evidenced by stability indices and sway characteristics. The LBP group demonstrated decreased postural stability during the second trial in the eyes-open condition, highlighting deficits in neuromuscular coordination compared to control subjects. Increased sway in the AP direction during the first trial and in both the ML and AP directions during the third trial further underscores specific deficits in lateral stability under eyes-open conditions as well as deficits in trunk proprioception and momentum control, which contribute to instability and fall risk.

These findings emphasize the complexity of human laterality and performance asymmetry, which warrants targeted interventions to improve lateral stability and neuromuscular coordination. Effective rehabilitation strategies should focus on enhancing neuromuscular control, reducing dependence on visual feedback, and optimizing proprioceptive function to improve stability and minimize the need for compensatory limb contact. Contradictory findings from one report suggest that COP may remain consistent despite changes in external visual input due to compensatory adjustments and complex interactions between visual perception and postural stability [65]. However, the study’s results, derived from healthy university students, limit its applicability to individuals with LBP who experience unique challenges in maintaining stability.

Contralateral toe-touch duration during the second trial in the eyes-open condition highlights its role as a compensatory mechanism for postural instability in LBP. This suggests that visual input alone is insufficient for maintaining postural control in LBP, potentially due to sensory integration deficits or an overreliance on visual feedback. Incorporating visual feedback into rehabilitation strategies may enhance postural stability and help reduce fall risk, especially for tasks requiring intentional movement control [66]. Although the study did not exclude the potential confounding effects of any individual differences, compensatory movement patterns appear vital for reducing fear in eyes-open conditions, building confidence, and minimizing sway to prevent falls. Our findings also reveal the dynamic nature of postural adjustments, with visual input significantly influencing toe-touch durations and sway excursions across trials. The LBP group’s reliance on compensatory strategies during repeated tasks suggests neuromuscular coordination challenges or fatigue-induced instability. This evidence highlights the need for targeted interventions to enhance proprioceptive function, reduce compensatory behaviors, and address fall risk associated with lateral instability.

This study highlights the critical role of sensory integration and compensatory strategies in postural control among individuals with LBP, emphasizing the need for targeted rehabilitation to address fall risk. Our findings support previous evidence that balance control relies on the central nervous system’s ability to modulate positional fluctuations by adjusting the COP within safe boundaries [62,67]. Visual feedback emerged as a significant factor in maintaining postural stability, aligning with prior studies [62,68,69,70]. However, conflicting evidence regarding the clinical utility of unilateral standing balance assessments persists. Some studies report weak associations between LBP-related factors (e.g., pain severity, central sensitization) and postural stability [71], while another study reported the predictive value of one-leg standing tasks for fall risk [24].

The lack of detailed exercise histories in baseline assessments may partially explain these inconsistencies. Mixed findings have been reported regarding the impact of visual input on balance stability in LBP populations, with a high risk of bias linked to balance assessment methods [23]. Comprehensive baseline assessments, including levels of pain-related dysfunction and activity histories, are needed to address these limitations. Systematic reviews examining associations between balance and fall risk in older adults emphasize the need for comprehensive assessments of gait, balance, and mobility [72,73]. Contradictory findings from studies comparing postural sway parameters in pain-free controls, individuals with LBP, and those with multisite musculoskeletal pain suggest that additional factors, including activity histories and pain-related dysfunction, must be accounted for in future research to clarify these relationships and improve rehabilitation strategies.

Our findings enhance clinical understanding of ML sway control and highlight the importance of optimizing sway on the non-dominant limb to achieve efficient postural stability. Anticipatory postural adjustments, which involve coordinated muscle activation to limit sway, play a crucial role in unilateral tasks [74]. Effective postural control also requires optimal distribution of somatosensory resources, which older adults with LBP may achieve through adaptive strategies [75]. Our findings align with the “lower limb laterality strategy”, where contralateral toe-touches address balance deficits and refine motor control [76]. This strategy highlights the instinctive engagement of the opposite limb to maintain stability during unilateral tasks. Similarly, the “tight control strategy”, which minimizes spinal movement and promotes co-contraction of torso and lower limb muscles, can enhance stability and reduce fall risk [77,78]. For individuals with LBP, this strategy helps manage instability and abnormal motor patterns, reducing the risk of falls and injury.

Our study protocol, which included repeated standing trials, provides insights into adaptive strategies that may reduce fall incidence in individuals with LBP. These findings align with research on lumbar proprioception and proprioceptive reweighting, where the central nervous system reallocates focus on proprioceptive inputs to maintain equilibrium [22,79,80,81]. Strengthening neuromuscular control and optimizing visual feedback utilization are critical for improving postural stability and reducing fall risk [42]. These results point to the critical need for interventions that aim to strengthen neuromuscular control and optimize the use of visual feedback. By focusing on these areas, therapeutic approaches can potentially enhance balance stability and reduce fall risk in the LBP group.

Several limitations may have influenced our findings, including cumulative fatigue, trial duration, anthropometric differences, and the diverse etiologies of LBP within our study population. While our study primarily focused on sensory integration deficits, it is important to consider that sway excursions may affect unilateral standing strategies, potentially increasing reliance on hip strategies for balance control. Future studies on postural stability in individuals with LBP should utilize a more comprehensive research design to identify specific patterns of postural control variability, explore neuromuscular adaptations, and incorporate detailed histories of exercise and physical activity. Our findings underscore the critical role of visual input, proprioceptive function, and compensatory strategies in maintaining balance in individuals with LBP, providing valuable insights for targeted rehabilitation interventions. Specifically, cognitive approaches may effectively address FOM by utilizing graded exposure and cognitive strategies to reduce maladaptive compensatory motions and promote adaptive movement patterns. In addition, targeted rehabilitation strategies that focus on improving sensory integration and enhancing neuromuscular control can help improve balance stability, decrease reliance on compensatory mechanisms, and enhance functional mobility, ultimately reducing the risk of falls.

## 5. Conclusions

The LBP group demonstrated significant balance deficits during repeated one-leg standing tasks, particularly under eyes-open conditions, with increased reliance on compensatory strategies like contralateral toe-touches. These findings highlight sensory integration and neuromuscular coordination impairments. Future studies should explore targeted interventions to enhance stability, reduce compensatory behaviors, and improve neuromuscular control in individuals with LBP.

## Figures and Tables

**Table 1:** Summary of subject anthropometric variables and measurements between groups.

Variables	Control Group	LBP Group	Statistics	*p*
Gender (Female/Male)	40 (19/21)	32 (20/12)	χ^2^ = 1.61	0.21
Age (years)	65.25 ± 9.22	68.28 ± 8.10	t = −1.46	0.15
BMI (kg/m^2^)	24.81 ± 5.32	23.64 ± 6.08	t = 0.86	0.39
ODI (%)	3.43 ± 3.53	26.69 ± 13.92	t = −10.19	0.001 **
VAS (1–10)	1.62 ± 1.34	4.24 ± 1.79	t = −7.08	0.001 **

LBP: low back pain, BMI: body mass index, ODI: Oswestry disability index, VAS: visual analog scale, ** *p* < 0.01.

## Data Availability

The data that supported the findings of this study are available from the corresponding author upon request.

## Abbreviations

The following abbreviations are used in this manuscript:

LBP	low back pain
BMI	body mass index
ODI	Oswestry disability index
VAS	visual analog scale
COP	center of pressure
ML	mediolateral
AP	anteroposterior
T	one-leg standing trial
TIB	time-in-boundary
EO	eyes-open condition
EC	eyes-closed condition
